# Supplementary material for: Magnetic Nanoparticles with Fe-N and Fe-C Cores and Carbon Shells Synthesized at High Pressures
Source: Materials (Basel). 2023 Nov 7;16(22):7063. doi: 10.3390/ma16227063 (PMC10672696; doi:10.3390/ma16227063)
Supplement: Supplementary file 1 [file materials-16-07063-s001.zip › materials-2673482-supplementary.pdf]

Supplementary

Magnetic Nanoparticles with Fe-N and Fe-C Cores and Carbon Shells Synthesized at High Pressures

Rustem H. Bagramov [1,*], Vladimir P. Filonenko [1], Igor P. Zibrov [1], Elena A. Skryleva [2], Boris A. Kulnitskiy [3], Vladimir D. Blank [3] and Valery N. Khabashesku [4,*]

[1] Vereshchagin Institute of High Pressure Physics, Russian Academy of Sciences, Troitsk, Moscow 108840, Russia; filv@hppi.troitsk.ru (V.P.F.); zibrov@hppi.troitsk.ru (I.P.Z.)
[2] Department of Materials Science of Semiconductors and Dielectrics, National University of Science and Technology MISiS, Moscow 119049, Russia; easkryleva@gmail.com
[3] Technological Institute for Superhard and Novel Carbon Materials, Troitsk, Moscow 108840, Russia; boris@tisnum.ru (B.A.K.); vblank@tisnum.ru (V.D.B.)
[4] Department of Materials Science and Nanoengineering, Rice University, Houston, TX 77005, USA
* Correspondence: bagramov@hppi.troitsk.ru (R.H.B.); khval@rice.edu (V.N.K.); Tel.: +7-495-851-0810 (R.H.B.); +1-713-409-9206 (V.N.K.)

Supporting Information

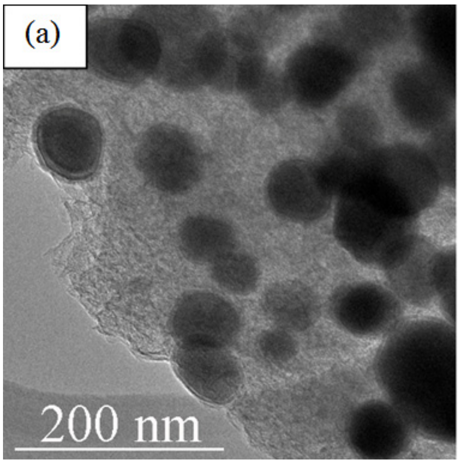

(a)

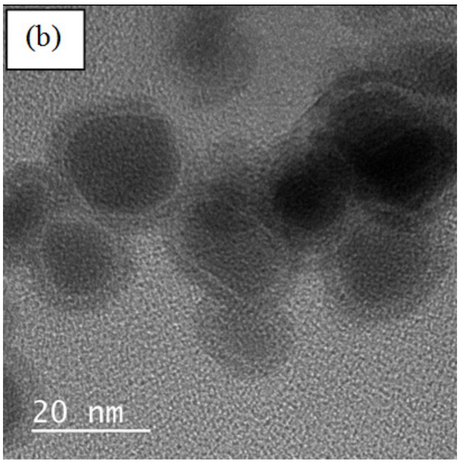

(b)

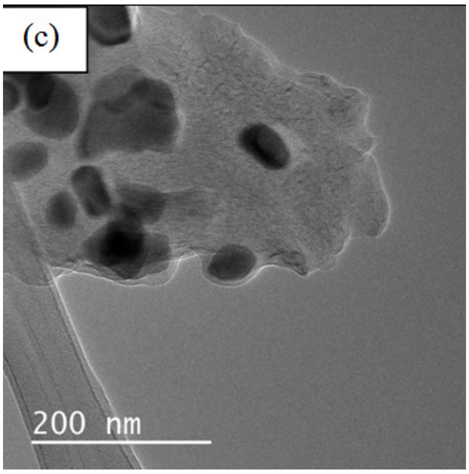

(c)

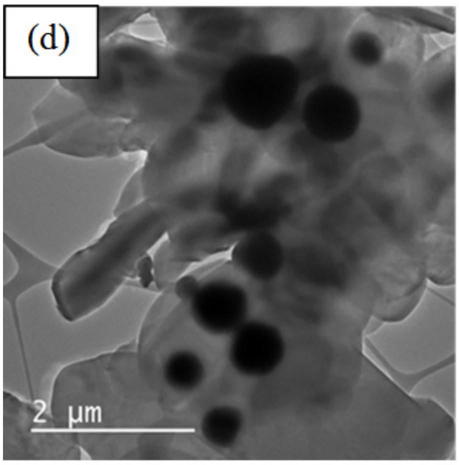

(d)

Figure S1. TEM images of nanoparticles: (a) obtained from ferrocene at 2 GPa/800ºC/30 min; (b) obtained from ferrocene at 8 GPa/800ºC/30 min; (c) obtained from ferrocene/melamine mixture at 8GPa/800ºC/30 min; (d) obtained from ferrocene/melamine mixture at 8GPa/800°C/0.5min + 8GPa/1250°C/0.3min).

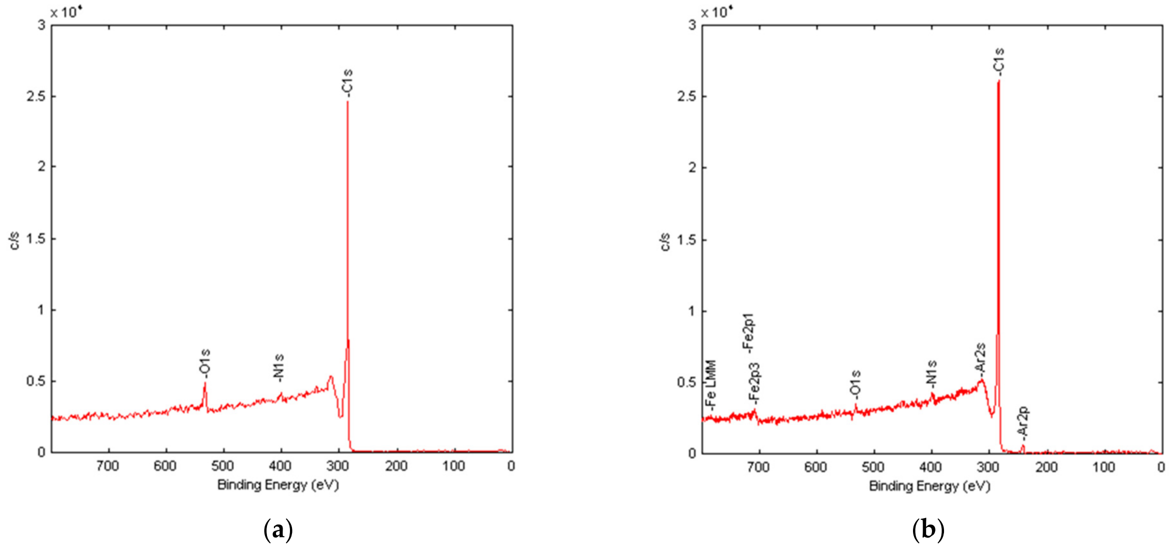

Figure S2. Survey XPS spectra for FerMelam/4GPa/800°C/30min sample taken before (a) and after (b) surface etching.
